# Supplementary material for: Obesity dysregulates feeding-evoked response dynamics in hypothalamic satiety neurons
Source: bioRxiv. 2025 May 27:2025.05.22.655553. Preprint. [Version 1] doi: 10.1101/2025.05.22.655553 (PMC12154894; doi:10.1101/2025.05.22.655553)
Supplement: Supplement 1 [file NIHPP2025.05.22.655553v1-supplement-1.pdf]

bioRxiv preprint doi: https://doi.org/10.1101/2025.05.22.655553; this version posted May 27, 2025. The copyright holder has placed this preprint (which was not certified by peer review) in the Public Domain. It is no longer restricted by copyright. Anyone can legally share, reuse, remix, or adapt this material for any purpose without crediting the original authors.

SUP. FIGURE 1

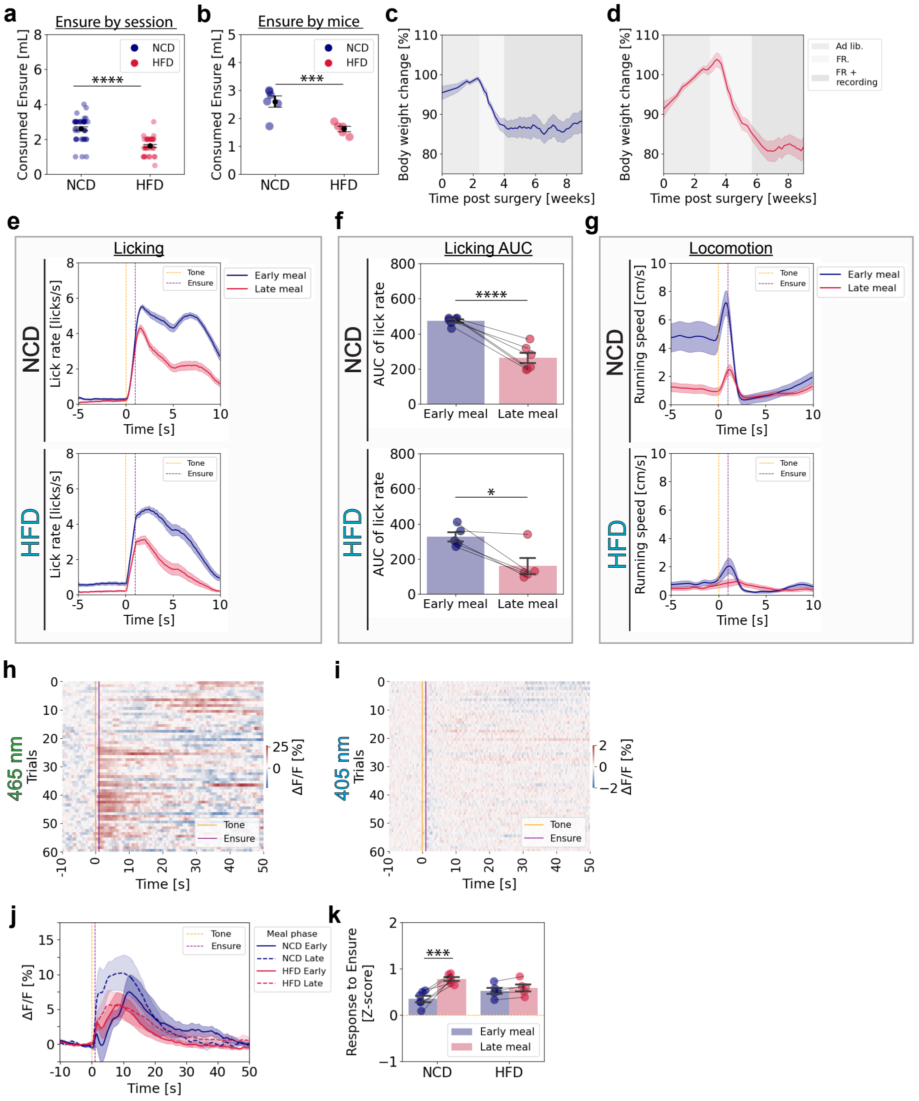

Supplementary Figure 1. Validation of GCaMP6s photometry signals and behavioral dynamics during food restriction in NCD- and HFD-fed mice

a, b, Total Ensure consumption during the individual photometry recording sessions (**a**) and across individual mice (**b**) for NCD-fed and HFD-fed mice.

c, d, Post-surgical bodyweight dynamics during ad libitum feeding (light gray), food restriction (FR, no shading), and continued food restriction maintenance during the photometry recording (FR + recording, dark shading) of NCD-fed (**c**) and HFD-fed (**d**) mice.

e, Mean lick rate during early and late meal phases for NCD-fed (top panel) and HFD-fed (bottom panel) mice.

bioRxiv preprint doi: https://doi.org/10.1101/2025.05.22.655553; this version posted May 27, 2025. The copyright holder has placed this preprint (which was not certified by peer review) in the Public Domain. It is no longer restricted by copyright. Anyone can legally share, reuse, remix, or adapt this material for any purpose without crediting the original authors.

f, Area under the curve (AUC) quantification of post-cue lick-rate, normalized to baseline pre-tone lick rate, for early and late meal phases of individual NCD-fed (top panel) and HFD-fed (bottom panel) mice.

g, Mean running during the early and late meal phase of NCD-fed (top panel) and HFD-fed (bottom panel) mice.

h, i, Representative heatmap of normalized ΔF/F GCaMP6s fluorescent responses detected using a 465 nm LED (**h**) and a 405 nm LED, reflecting movement artifacts and auto-fluorescence (**i**). Responses in each trial are normalized to the 10 s baseline before the cue onset. Note the range in **i** is smaller than in **a**.

j, Early and late meal responses to Ensure for NCD-fed and HFD-fed animals, expressed as ΔF/F.

k, Z-scored PVH^{MC4R} neuronal responses of each animal during early and late meal phases for all recordings in NCD-fed and HFD-fed mice, averaged from 1 – 20 s post-cue presentation. NCD: 6 mice; HFD: 5 mice.

a, b, f, – unpaired, two-tailed Student t-test.

k, Two-way Anova with Tukey post hoc test, for Early vs. Late meal within each condition and early phases between conditions.

a – g, j – k, Data are represented as the mean ± s.e.m either as error bars (**a, b, f, k**) or as shaded area (**c, d, e, g, j**).

a, b, k, * - P <0.05, *** - P <0.001, **** - P <0.0001.

bioRxiv preprint doi: https://doi.org/10.1101/2025.05.22.655553; this version posted May 27, 2025. The copyright holder has placed this preprint (which was not certified by peer review) in the Public Domain. It is no longer restricted by copyright. Anyone can legally share, reuse, remix, or adapt this material for any purpose without crediting the original authors.

SUP. FIGURE 2

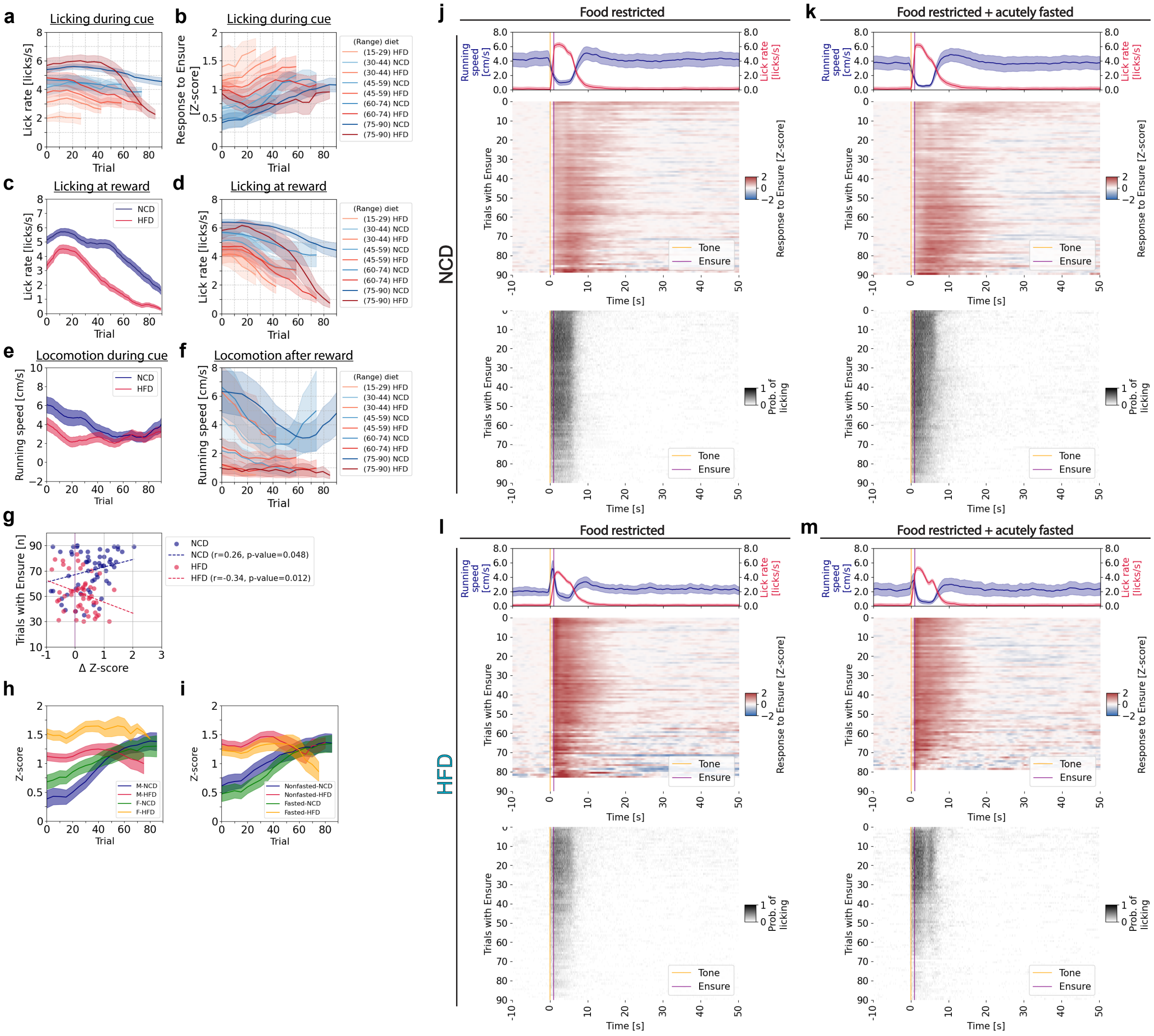

Supplementary Figure 2. Diet- and fasting-dependent modulation of PVH^{MC4R} neuronal activity, licking behavior, and trial engagement in NCD- and HFD-fed mice

a, Lick rate during the 1 s cue window grouped by the number of triggered trials (ranges: 15–29, 30–44, 45–59, 60–74, 75–90) in NCD-fed (blue shade) and HFD-fed (red shade) mice.

b, Single trial mean neuronal responses 1-9 s post cue (peri-licking) grouped by the number of triggered trials (ranges: 15–29, 30–44, 45–59, 60–74, 75–90) in NCD-fed (blue shade) and HFD-fed (red shade) mice.

bioRxiv preprint doi: https://doi.org/10.1101/2025.05.22.655553; this version posted May 27, 2025. The copyright holder has placed this preprint (which was not certified by peer review) in the Public Domain. It is no longer restricted by copyright. Anyone can legally share, reuse, remix, or adapt this material for any purpose without crediting the original authors.

c, Single trial mean licking rate during the reward period (1 – 9 s post-cue) across 90 recorded trials from NCD-fed and HFD-fed groups (minimum 30 successfully triggered trials).

d, Single trial mean licking rate during the reward (1 – 9 s post cue) across 90 recorded trails grouped by the number of triggered trials (ranges: 15–29, 30–44, 45–59, 60–74, 75–90) for NCD-fed (blue shading) and HFD-fed (red shading) mice.

e, Single trial mean running speed across 90 recorded trails from NCD-fed and HFD-fed mice (minimum 30 successfully triggered trials).

f, Single trial mean running speed during the pre-cue period across 90 recorded trails, grouped by the number of triggered trials (ranges: 15–29, 30–44, 45–59, 60–74, 75–90) for NCD-fed (blue shade) and HFD-fed (red shade) mice.

g, Number of triggered trials out of the 90 possible trials recording session versus ΔZ-score between computed by subtracting the mean early meal response from the mean late meal response. The dashed line represents the linear relationship between meal size (triggered trials with Ensure) and the ΔZ-score (i.e. the change in response to Ensure from early to late in the meal).

h, Single trial mean peri-licking PVH^{MC4R} responses (1 – 9 s post-cue) across 90 recorded trials from female (F) and male (M) NCD-fed and HFD-fed groups. Only recordings with at least 30 successfully triggered trials were included. M-NCD: 26 recordings / 3 mice; M-HFD: 31 recordings / 4 mice; F-NCD: 33 recordings / 4 mice; F-HFD: 23 recordings / 3 mice.

i, Single-trial mean peri-licking PVH^{MC4R} responses (1 – 9 s post-cue) across 90 recorded trials for food restricted (nonfasted) and food restricted plus acute fasting (fasted) NCD-fed and HFD-fed groups. Only recordings with at least 30 successfully triggered trials were included.

j – m, Heatmaps summarizing GCaMP6s photometry signals (top panel) and licking behavior (bottom panel) from PVH^{MC4R} neurons in food-restricted-only session (**j,l**) and in food restricted-plus acutely-fasted sessions (**k, m**) for NCD-fed (**j, k**) and HFD-fed (**l, m**) animals. Mean running speed and licking rates across all triggered trials are shown above the heatmaps. Only recordings with a minimum of 30 successfully triggered trials were included. Trial structure: 10 s baseline before cue (tone) onset (t = 0 s), followed by Ensure delivery at t = 1 s.

a – e, Nonfasted NCD: 31 recordings / 7 mice (**i, j**); Food restricted plus acutely fasted NCD: 28 recordings / 7 mice (**i, k**); Nonfasted HFD: 31 recordings / 7 mice (**i, l**); Food restricted plus acutely fasted HFD: 23 recordings / 7 mice (**i, m**).

e – m, Data are represented as the mean ± s.e.m. * - P <0.05, *** - P <0.001, **** - P <0.0001.

c, e, g, NCD: 59 recordings / 7 mice; HFD: 54 recordings / 7 mice.

bioRxiv preprint doi: https://doi.org/10.1101/2025.05.22.655553; this version posted May 27, 2025. The copyright holder has placed this preprint (which was not certified by peer review) in the Public Domain. It is no longer restricted by copyright. Anyone can legally share, reuse, remix, or adapt this material for any purpose without crediting the original authors.

a, b, d, f, Recordings for each meal size range: NCD (15–29): 0; HFD (15–29): 6; NCD (30–44): 6; HFD (30–44): 13; NCD (45–59): 10; HFD (45–59): 23; NCD (60–74): 13; HFD (60–74): 7; NCD (75–90): 28; HFD (75–90): 4.

bioRxiv preprint doi: https://doi.org/10.1101/2025.05.22.655553; this version posted May 27, 2025. The copyright holder has placed this preprint (which was not certified by peer review) in the Public Domain. It is no longer restricted by copyright. Anyone can legally share, reuse, remix, or adapt this material for any purpose without crediting the original authors.

SUP. FIGURE 3

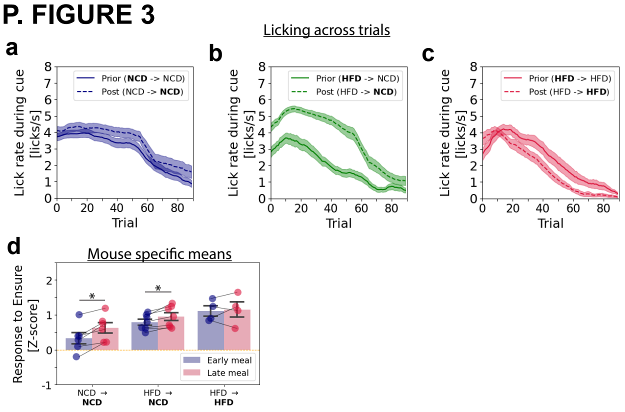

Supplementary Figure 3. Impact of diet switch on cue-evoked licking behavior and PVH^{MC4R} dynamics during early and late meal phases

a – c, Single-trial mean lick rate during cue presentation (0 – 1 s post-cue onset) across 90 trials averaged across recordings with at least 30 successfully triggered trials in the NCD to NCD (**a**), HFD to NCD (**b**), and HFD to HFD (**c**) groups, comparing pre-diet-switch (solid line) and post-diet-switch (dashed line) conditions.

a – c, Recording and animal numbers during exposure to the first diet: **NCD** to NCD: 44 recordings / 6 mice; **HFD** to NCD: 52 recordings / 7 mice; **HFD** to HFD: 35 recordings / 4 mice. Recording and animal numbers during exposure to the second diet: NCD to **NCD**: 43 recordings / 6 mice; HFD to **NCD**: 42 recordings / 7 mice; HFD to **HFD**: 23 recordings / 4 mice.

d, Mean Z-scored PVH^{MC4R} neuronal responses during early (first 15 trials) and late meal (last 15 trials) phases of the meal, for individual mice (averaged across sessions per mouse) across the following conditions post-diet switch: NCD to NCD, HFD to NCD, HFD to HFD. Paired, two-tailed t-test between early-meal and late-meal trials.

a – d, Data are represented as the mean ± s.e.m across sessions (**a – c**) or across mice (**d**). * - P <0.05, ** - P <0.01, *** - P <0.001.
